# Revision of the Retracted Posttracheostomy Scar by Anatomical Restoration; Four Layer Closure

**DOI:** 10.1055/s-0044-1787294

**Published:** 2024-06-19

**Authors:** Jaeyoung Cho, Jimin Lee, Sang Yoon Kang

**Affiliations:** 1 Department of Plastic Surgery, Kyung Hee University Hospital, Seoul, Korea; 2 Department of Plastic Surgery, College of Medicine, Kyung Hee University Hospital, Seoul, Korea

**Keywords:** tracheostomy, scar, retraction, fistula, adhesion, restoration

## Abstract

Most tracheostomy scars are depressive and adherent to the underlying trachea, which causes up and down movement when swallowing. This tracheocutaneous tethering causes discomfort, pain, dysphagia, and bad appearance. A tracheocutaneous fistula may be accompanied. Here, we present a new method for reconstructing a tracheostomy scar deformity with tracheocutaneous tethering: layer-by-layer restoration of the anatomical structure with a subcutaneous fat tissue blanket. The scar tissue was fully excised, with the associated skin and subcutaneous tissue. The bilateral strap muscles around the scar were dissected proximally and distally and approximated to the midline, secured without tension. Bilateral platysma muscle flaps cover them firmly. The subcutaneous tissue around the incision margin, which included the superficial cervical fascia was elevated to form a fat blanket, closed transversely. The skin was closed after confirming the absence of retraction upon swallowing. From 2010 to 2018, 10 patients with tracheocutaneous tethering and one patient with tracheocutaneous fistula underwent surgery. All patients were functionally and aesthetically satisfied with the results. The only complication was a hypertrophic scar in one patient, which was managed with a triamcinolone injection. An anatomical layer-by-layer restoration with a fat blanket provided consistent, satisfying results for correcting tracheostomy scar deformities without using additional tissue. This simple method was effective for reconstructing tracheocutaneous tethering.

## Introduction

Tracheostomy is one of the most commonly performed procedures for airway control in intensive care units. In prolonged endotracheal intubations, a tracheostomy can reduce the need for sedation and improve patient comfort. Percutaneous tracheostomy is currently a common method for securing the airway, because compared with a standard tracheostomy, it can be performed bedside with a smaller workforce, more cost-effective, and less tissue is injured. Despite these advantages, late complications may occur after the procedure, such as tracheal stenosis, voice changes, retraction of the scar, and tracheal fistula. 1 2

After decannulation, tracheostomy site heals with secondary intention. The space resulting after decannulation is filled with granulated tissue. Subcutaneous tissue defect and wound contraction result in a depressive scar, and subsequent wound contracture results in tracheocutaneous tethering. Subsequent tissue atrophy and defect worsens the tethering. Additionally, when the skin adheres to the tracheal wall through penetrating scar tissue during healing, the skin in front of the trachea moves with tracheal movement. This retraction causes unpleasing appearance, discomfort, pain, and dysphagia. 3 4 Furthermore, when the epithelium grows into the tracheostomy site during healing, a tracheocutaneous fistula can occur. In the presence of a fistula, patients are susceptible to excessive tracheal secretion, dysphagia, and phonatory difficulties. 5

A tracheostomy scar revision is commonly performed to improve the functional and aesthetic aspects. The first goal of this procedure is to remove the tracheocutaneous tethering or fistula, and prevent recurrence. The second goal is to eliminate surrounding depression and recover a natural neck contour. Various methods have been described to achieve these goals, including muscle transposition 3 6 7 8 9 and an autologous or biomaterial graft transplantation. 4 5 10 11 12

In the neck anatomy, layers of muscles and soft tissue around the tracheostomy site are accessible for tracheostomy scar correction. 13 14 15 The infrahyoid muscles (strap muscles) are located deeply over the anterior tracheal wall, and the platysma muscles exist superficially. The superficial cervical fascia, which is a continuous sheath of fibrofatty subcutaneous tissue, encloses the platysma muscle from above. Using the muscles and superficial cervical fascia over the tracheal wall, we developed a simple and effective method for reconstructing tracheostomy scar deformities without the use of extraneous tissue grafts. Here, we present this novel method utilizing layer by layer restoration of anatomical structures and a fat blanket.

## Ideas

### Materials and Methods

From 2010 to 2018, 11 patients (mean age 37 years; range 14–64 years) underwent surgery for a tracheostomy scar deformity. The tracheostomies had been performed during intensive care, due to head trauma ( *n* = 7), acute respiratory arrest ( *n* = 2), axonal motor neuropathy in Guillain–Barre syndrome ( *n* = 1), and head and neck oncological surgery ( *n* = 1). The tracheostomy was maintained for an average of 4.8 months (range 14 days–26 months). Revision surgery was performed after an average interval of 31.9 months (range 2 months–14 years) after removing the tracheostomy tube. All patients had tracheocutaneous tethering, and one patient also had a tracheocutaneous fistula. All surgeries were performed by a senior surgeon, who restored the anatomical structure layer-by-layer with a fat blanket ( Table 1 ). Two patients; 18- and 38-year-old (both male) were smokers, and the rest were nonsmokers. One patient who had received tracheostomy due to head and neck oncology had not received previous radiotherapy or lymph node dissection. All informed consents were obtained via the Institutional Review Board of our institution (IRB No. 2020-11-018).

**Table 1:** Patient demographics, reasons requiring tracheostomy, tracheostomy cannula duration and interval, follow-up period, results, and recurrence

Patient number	Gender/Age	Reasons requiring tracheostomy	Duration of tracheostomy cannula	Time from cannula removal to surgery	Follow-up period	Result	Recurrence
1	F/22	Respiratory arrest (after mandible angle resection and rhinoplasty)	6 mo	8 mo	5 mo	Good	No
2	M/45	Head trauma (brain hemorrhage and C3, 4 fracture)	5 mo	1 yr 2 mo	10 mo	Good	No
3	M/64	Head trauma (brain hemorrhage from pedestrian TA)	1 mo	8 mo	7 mo	Good	No
4	M/18	Head trauma (brain hemorrhage from motorcycle TA)	3 mo	3 yr	8 mo	Good	No
5	F/30	Head trauma (pontine hemorrhage from arteriovenous malformation)	14 d	2 mo	7 mo	Good	No
6	F/50	Head trauma (aneurysm rupture)	2 mo	8 mo	8 mo	Good	No
7	F/14	Head trauma (brain hemorrhage from pedestrian TA)	2 mo	6 mo	10 mo	Good	No
8	F/59	Head and neck oncology surgery (thyroid cancer thyroidectomy)	5 mo	6 yr	11 mo	Good	No
9	M/38	Respiratory arrest (pneumonia)	2 mo	9 mo	3 mo	Good	No
10	M/42	Axonal neuropathy (Guillain–Barré syndrome)	3 mo	1 yr 7 mo	18 mo	Good, hypertrophic scar	No
11	M/21	Head trauma (brain hemorrhage from arteriovenous malformation)	26mo	14 yr	22 mo	Good	No

Abbreviations: d, day; mo, month; TA, traffic accident; yr, year.

### Surgical Technique



**Video 1** Fat blanket identification and approximation. Fat blanket, which is a superficial cervical fascial flap including fat tissue between the platysma muscle and the skin, was identified and approximated to the midline, secured with an interrupted absorbable suture.


**Fig. 1:**
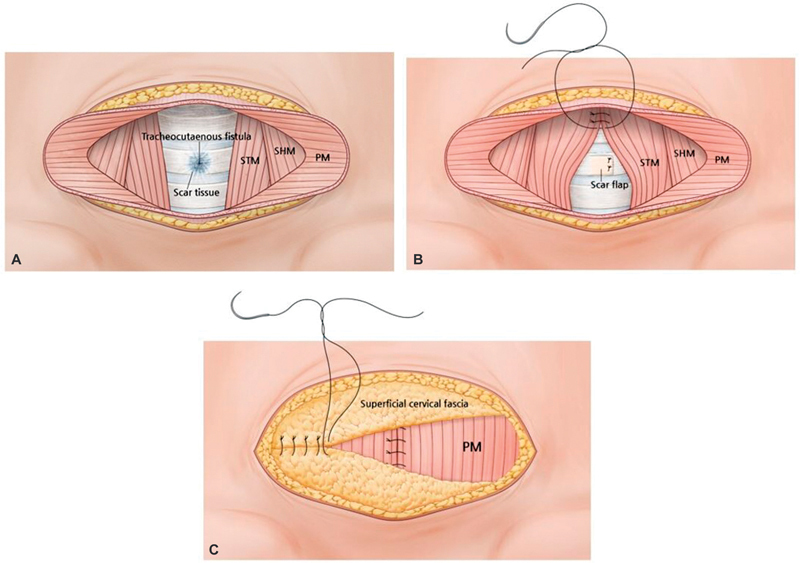
Layer-by-layer restoration technique after tracheostomy scar excision. ( **A** ) Scar tissue was excised completely. In case of a tracheocutaneous fistula, the fistula was sealed off with nearby scar tissue flap. ( **B** ) Bilateral strap muscle (sternothyroid muscles and sternohyoid muscles) were dissected, elevated, and approximated to the midline in order. Bilateral platysma muscles were dissected, raised, and medially approximated over the strap muscles. ( **C** ) The superficial cervical fascial flap, including subcutaneous fat tissue, was secured at the midline transversally over the plastysma muscle. PM, platysma muscle; SHM, sternohyoid muscle; STM, sternothyroid muscle.

**Fig. 2:**
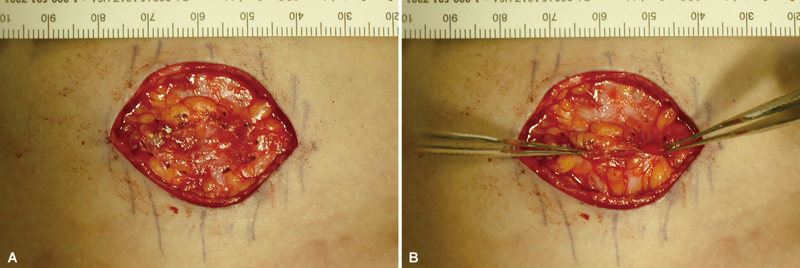
A superficial cervical fascial flap, including fat tissue, was advanced to the midline. The absence of skin retraction and tightness were checked before closure. ( **A** ) Black dotted line indicates the transverse medial edges of the elevated adipofascial flaps. ( **B** ) Adipofascial flaps are brought to the horizontal midline with Addison's forceps.

Each patient was placed in a supine position, and the procedure was conducted under local anesthesia. The tracheostomy scar and retracted area were marked with gentian violet ink in an elliptical shape, along the natural skin fold. Normal skin around the tracheostomy scar was preserved as much as possible to achieve minimal tension in the skin closure and good long-term results. For dissection, the depth of the incision at the trachea reached down to the root of the scar tissue, but avoided tracheal injury. Then the scar tissue was excised completely. When the fistula was confirmed preoperatively, a few millimeters of the scar tissue attached to the anterior trachea were left to serve as scar-tissue flaps ( Fig. 1 , *top left* ). These elevated flaps were turned over, invaginated into the fistula, with the more superficial layers newly placed inside the fistula on the tracheal surface. Monofilament, multibraided, absorbable suture (Vicryl 4–0 and 5–0) was used for interrupted suture or purse-string suture ( Fig. 1 , *top right* ). A simple air bubble test with saline was performed to detect air leakage that may occur during breathing. Strap muscles (sternohyoid and sternothyroid muscles) around the trachea were identified and dissected in order. The muscles were carefully approximated to the midline, and sutured securely with monofilament, multibraided, absorbable suture (Vicryl 4–0 and 5–0; Fig. 1 , *top right* ). We have used the same size sutures for all muscle and fat layers. Next, bilateral platysma muscles were identified and dissected laterally from the overlying subcutaneous fat tissue and underlying strap muscles. Platysma muscle repair was done close to the midline ( Fig. 1 , *bottom* ). The superficial cervical fascia with adipose tissue was dissected and mobilized to the horizontal midline to “make a fat blanket,” with no or minimal tension. The superficial cervical fascial flap was mobilized to the midline and secured ( Fig. 1 , *bottom* and Fig. 2 ; see Video 1 , which demonstrates fat blanket identification and approximation to midline). After closure and coverage with superficial cervical fascial flap, patient was asked to swallow. Free movement with no scar tissue attachment upon swallowing was confirmed in the surgical field, and skin was closed in two layers: absorbable suture (Vicryl, 5–0) was used for the dermal closure, and nylon suture (Nylon, 6–0) or skin glue for skin closure. No drain was inserted.

There was no special care to prevent relapse of retraction. Regular, routine postoperative wound management and scar care was done. This included daily saline cleansing of the wound, application of antibiotic ointment, and coverage with foam dressing. After all stitches were removed, a routine scar management was followed, which included using products such as silicone gel or silicone sheets.

### Results



**Video 2** Before and after tracheostomy scar revision surgery in a 21-year-old male patient. Tracheocutaneous tethering observed. Three months after the layer-by-layer reconstruction with a fat blanket, the depression and retraction were absolutely resolved, and the natural neck contour was recovered.


**Video 3** Before and after tracheostomy scar revision surgery in a 38-year-old male patient. Tracheocutaneous tethering and fistula were observed. One year after the layer-by-layer reconstruction with a fat blanket, the depression, retraction, and fistula were completely resolved, and the natural neck contour was recovered with an inconspicuous linear scar.


**Fig. 3:**
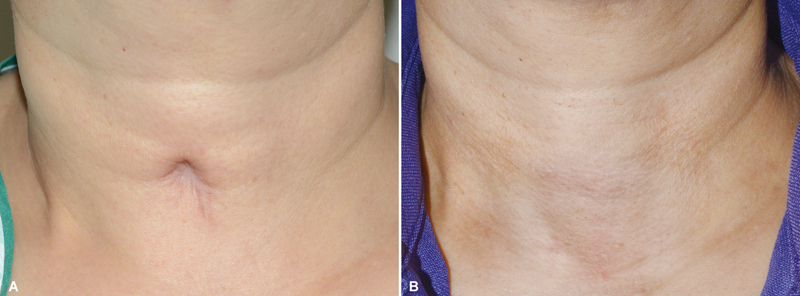
Pre- and postoperative photos of a tracheostomy scar revision surgery in a 50-year-old female patient. ( **A** ) Tracheocutaneous tethering was noted on the neck. Tracheostomy had been performed during aneurysmal rupture repair surgery, and was maintained for 2 months. Eight months after removal of the tube, scar revision was performed. ( **B** ) Ten months postoperatively. A natural neck contour is recovered with absolute resolution of the retraction, and an inconspicuous scar.

**Fig. 4:**
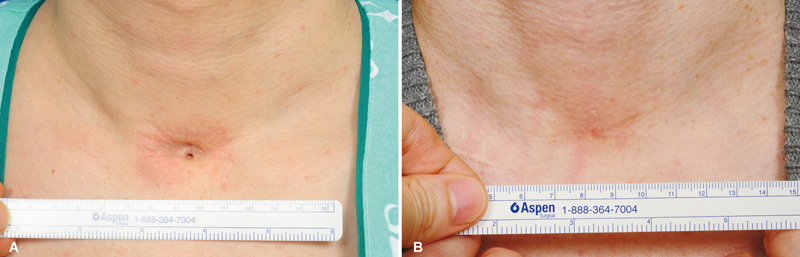
Pre- and postoperative photos of a tracheostomy scar revision surgery in a 38-year-old male patient. ( **A** ) Tracheocutaneous tethering with fistula. Tracheostomy had been performed to treat acute respiratory failure due to pneumonia, and was maintained for 2 months. Nine months after removal of the tube, scar revision and repair of the tracheocutaneous fistula were performed. **(B)** One year postoperatively. Depression and fistula are completely resolved, and a natural neck contour is recovered with an inconspicuous linear scar.

The results of the procedures were satisfactory, with a mean follow-up of 10 months (range 5–22 months; Table 1 ). In all patients, the depression was corrected, the retraction disappeared, and the tracheocutaneous fistula was eliminated. A natural neck contour was restored in all patients. No complications occurred, such as infection, hematoma, recurrence of a retraction, or fistula. In one case, a hypertrophic scar occurred, despite postoperative scar management with silicone gel and a sheet. This scar was treated with an intralesional triamcinolone (10 mg/mL) injection. The other patients, the postoperative scars were nearly inconspicuous, hidden by the natural neck skin fold (see Video 2 , which demonstrates the before and after tracheostomy scar revision surgery in a 21-year-old male patient; see Video 3 , which demonstrates the before and after tracheostomy scar revision surgery in a 38-year-old male patient, and Figs. 3 and 4 ).

## Discussion

We reconstructed the soft tissue defect over the trachea while revising tracheostomy scar deformities. Three tissue layers were inserted between the skin and the trachea, including the strap muscles, the platysma muscles, and the superficial cervical fascial flap, to form a fat blanket. The strap muscles and platysma muscles, located above the tracheostomy site, are well-developed and easily detached from the surrounding tissue. The superficial cervical fascia, a translucent membrane above the platysma muscle that contains adipose tissue, 15 were also readily dissected and advanced to the horizontal midline in the same surgical field.

Tracheostomy is a relatively less destructive procedure; therefore, each structure was well maintained, even after the long-term tube insertion and contracture. We found the muscles and fatty layer around the tracheostomy space to be intact. When excising scar tissue, however, the surgeon should aim to conserve as much of the normal tissue as possible. Tension between the tissues might split the muscle and fat blanket structures, which would lead to revision failure. Additionally, skin tension causes scar widening.

To date, several methods have been described for correcting tracheostomy scar deformities with retraction. To correct depression, local muscles, such as the strap muscles, sternocleidomastoid muscles, or platysma muscles, have been advanced, transposed, split, or turned over. 3 6 7 8 9 To prevent adhesion, autologous or biomaterial grafts have been used as an interposition graft. 4 5 10 11 12 There are several types of biomaterial grafts. For example, Carlson et al 10 sutured the strap muscles medially and placed allogeneic dura over the sutured muscles to prevent skin adhesion to the sutured muscle. Skigen et al 12 used Tutoplast-processed dura mater as a barrier membrane in a tracheostomy scar revision. Tollefson et al 4 performed tracheostomy scar revision by moving the strap muscles to the median line, then by covering the muscles with Alloderm® (LifeCell Corp, Branchburg, NJ). However, these biomaterial grafts have some disadvantages, such as additional cost, increased risks of viral transmission, and formation of seroma. 4

In contrast, autografts are typically made of fat tissues to create a relatively natural-looking neck. For example, Stanton et al 5 harvested a dermal fat graft from the abdomen and placed it over the infrahyoid muscle to prevent adhesion. Mazzola et al 11 performed autologous fat graft. After subcision of the fibrous band and scar tissue, fat harvested from the lower abdomen was injected to fill the depression defect and improve scar quality. However, the main disadvantage of an autograft is that it requires a secondary donor site.

In the present study, one lightweight patient did not have sufficient fat tissue in the neck. In this case, we inserted an allodermal matrix in the subcutaneous plane immediately superior to the sternal notch, and the result was acceptable. In other cases, the three-layer padding by placing the two strap muscles, platysma muscles, and fat blanket between the skin and the trachea was sufficient to fill the volume of the depression, and prevent adhesion.

Treating the tracheocutaneous fistula is challenging, as are other fistulas. Various closure methods have been described including secondary intention healing after chemical cauterization, primary closure, reconstruction with local flaps, muscle flaps, and free flaps. 16 17 18 Persistent tracheocutaneous fistulas are correlated with a cannulation period longer than 4 months, obesity, neck irradiation, infection, and nutritional deficits. 7 19 In our study, one patient presented with a tracheocutaneous fistula that had developed after 2 months of cannulation and persisted for 9 months after decannulation. Due to the relatively small size of the fistula, we chose to treat it by turning over a scar flap. This one-stage procedure provided a satisfactory result. The inner lining of the fistula tract became the inner surface of the trachea. This turnover technique is useful and straightforward, but this technique required secure suturing to prevent air leakage. Covering the scar flap with strap muscle flaps also reinforced airtightness.

The superficial cervical fascia with subcutaneous fat tissue, which we called a “fat blanket,” served as filler between the trachea and skin. Sufficient soft tissue volume was achieved with the superficial fatty fascia. In the neck, the majority of fat is located in the supraplatysmal plane. Nearly half (44.7%) of the fat in the neck is found in this superficial layer, rather than in the subplatysmal layer. 20 Medial placement of the superficial cervical fascia provided sufficient fat tissue to fill even severe depressions. Also, we have not experienced tension while performing the revision surgery. Because tracheostomy is not a defective procedure, by identifying the anatomical layers of muscles and soft tissue around the tracheostomy site, we were able to perform a tension-free revision. Moreover, atrophy and adhesion of the subcutaneous tissues to the tracheal wall is the main cause of tracheocutaneous tethering. 5 Therefore, restoring the subcutaneous tissue was a rational, anatomical approach for reconstructing the tracheostomy scar deformity.

The superficial cervical fascia is also an essential structure for maintaining the natural neck contour. It broadly encloses the platysma muscles, fuses with the deep fascia of the pectoralis and deltoid muscles inferiorly, and becomes the superficial musculoaponeurotic system above the jawline. 15 By advancing the superficial cervical fascia over the depression, a more natural, smooth neck contour can be obtained after scar revision.

Some factors act as barriers to a successful revision. The tracheostomy is typically performed in emergency conditions or very severe cases that require respiratory assistance. These patients must recover from serious surgeries or conditions, like neurosurgery, head and neck surgery, complex traumatic injury, or respiratory illness. 1 Such patients may not think ahead about undergoing another surgery to revise the tracheostomy scar, until their condition has improved sufficiently to allow social activity or until self-confidence has been restored. Moreover, the cost of revising a tracheostomy scar is not covered by the social services system, regardless of the presence of retraction or a tracheal fistula. For these reasons, this study included relatively few patients, and further studies with larger number of patients should follow.

Tracheostomy scars with retraction, and fistulas, can cause unaesthetic appearance, discomfort, pain, and dysphagia. Here, we described a method for revising tracheostomy scars and tracheocutaneous fistulas with an anatomical restoration by using the strap muscles, platysma muscles and the superficial cervical fascia with fat tissue. This method achieved a natural neck contour. No recurrence was noted, and no other complications occurred, except for one case of a hypertrophic scar. We conclude that an anatomical restoration with a fat blanket is a feasible reconstructive option and provides good result.
